# Program Implementers' Evaluation of the Project P.A.T.H.S.: Findings Based on Different Datasets over Time

**DOI:** 10.1100/2012/918437

**Published:** 2012-05-01

**Authors:** Daniel T. L. Shek, Cecilia M. S. Ma

**Affiliations:** ^1^Department of Applied Social Sciences, The Hong Kong Polytechnic University, Hong Kong; ^2^Public Policy Research Institute, The Hong Kong Polytechnic University, Hong Kong; ^3^Department of Social Work, East China Normal University, Shanghai 200241, China; ^4^Kiang Wu Nursing College of Macau, Macau; ^5^Division of Adolescent Medicine, Department of Pediatrics, Kentucky Children's Hospital, University of Kentucky College of Medicine, Lexington, KY 40506, USA

## Abstract

This paper integrates the evaluation findings based on program implementers in nine datasets collected from 2005 to 2009 (244 schools and 7,926 implementers). Using consolidated data with schools as the unit of analysis, results showed that program implementers generally had positive perceptions of the program, themselves, and benefits of the program, with more than four-fifths of the implementers regarding the program as beneficial to the program participants. The subjective outcome evaluation instrument was found to be internally consistent. Multiple regression analyses revealed that perceived qualities of the program and program implementers predicted perceived effectiveness of the program. In conjunction with evaluation findings based on other sources, the present study provides support for the effectiveness of the Tier 1 Program of the Project P.A.T.H.S. (Positive Adolescent Training through Holistic Social Programmes) in Hong Kong.

## 1. Introduction

In the context of evaluation, subjective outcome evaluation or the client satisfaction approach is a widely used strategy to evaluate programs in human services. There are several strengths of subjective outcome evaluation [1–3]. First, it is easy to administer and is low in cost. Second, it focuses on the subjective perception of the respondent, thus avoiding the criticism that evaluation methods are dominated by the views of the experts. Third, it does not require sophisticated statistical techniques in order to analyze the related data. Finally, with the use of validated measures of client satisfaction, there are findings suggesting that there is convergence between subjective outcome and objective outcome findings and thus indicates that subjective outcome can be regarded as a “proxy” for assessing the effectiveness of a program [4].

Traditionally, subjective outcome evaluation has been predominately used to understand the perceptions of program participants (i.e., clients who join the program). However, it is equally important to examine the view of the program implementers, especially those who are not directly involved in the development process of the program. It is quite common that youth programs, such as substance abuse and violence prevention programs, are often designed and developed by academics and experienced field workers but implemented by front-line workers in the field, such as teachers in school settings and social workers in social welfare settings. Facing programs with these characteristics, front-line workers might have strong resistance towards the program because they have had little involvement during the development process. Things get worse when they do not agree with the program philosophy and mission. Furthermore, rumors about additional workload and organizational constraints may adversely affect staff morale, which in turn lowers the workers' motivation to implement the program in an authentic and enthusiastic manner.

There are several reasons why subjective outcome evaluation should include the perceptions of the program implementers. First, because program implementers are also stakeholders of the developed programs, their views should be understood. According to the Joint Committee on Standards for Educational Evaluation [5], stakeholders should be identified (Standard U1) and their views should be taken into account (Standard F2). This is consistent with the framework of utilization-focused evaluation [6], which posited that relevant stakeholders should also be involved in the evaluation process. From the interpretive and constructivist perspectives, as the reality is fluid, it is important to look at the experiences of different stakeholders. Politically and practically speaking, collecting views from the program implementers can definitely give a more balanced view about the program effect and thereby facilitate the program implementation process.

Second, as program implementers are usually more experienced than the clients, it can be argued that their views may be more “accurate” than those of the clients. For example, in adolescent prevention programs, it is common to ask the program participants and workers regarding their perceptions of the program design, objectives, and rationales. It seems that the program implementers in this context possess better skills and experience in judging the quality of the program designed. Similarly, with their professional training and experience, workers will be in a better position to assess the effectiveness of the program and they can view the program from a deeper perspective.

Third, it can be argued that subjective outcome evaluation based on the perspective of the worker would facilitate reflective practice. Osterman and Kottkamp [7] noted that professionals' needs and desires for feedback about their own performance and personal reflections can lead to professional growth and development. Similarly, Taggart and Wilson [8] highlighted the role of reflective practice in teaching. Because reflective practice has become more critical in different disciplines, such as education and social work, the practice of subjective outcome evaluation can help professionals to reflect on the program they delivered and to assess their input and quality of the implementation. In short, subjective outcome evaluation based on the perspective of the workers can facilitate reflective practice among program implementers.

Fourth, the inclusion of subjective outcome evaluation based on the worker's perspective can give them a sense of fairness, which is an important determinant of the morale of the workers. Obviously, if only the program participants are asked to assess the program implementers, the workers may think the evaluation is rather unfair because only the voices of the program participants are heard. Furthermore, when the workers are invited to express their views and thoughts freely, they will feel more respected and less likely to view themselves as the victims of consumerism.

Fifth, in situations where a developed program is used in different sites (e.g., school-based positive youth development programs), implementation experiences may vary across schools. For some sites where the implementation experiences are negative, such news may spread quickly and the related rumors may adversely affect the program. Nevertheless, if the researchers can build a systematic profile of the experiences of the workers for documenting and disseminating the related findings, this can dispel the rumors and provide an accurate picture that truly reflects the implementation quality. In short, evaluation based on the program implementers can provide a better view about the implementation process.

Finally, according to the principle of triangulation, collection of subjective outcome evaluation data from different data sources can definitely help to answer the question of whether data collected from different sources generate the same picture. For example, while the workers may perceive themselves as performing well during the implementation process, the students may not necessarily have the same perceptions. Similarly, students and instructors may have different views on the students' learning motivation. In short, inclusion of subjective outcome evaluation data from different perspectives can enable researchers to grasp a more complete and balanced picture regarding perceived program attributes and effects.

Reppucci [9] also indicated that intervention programs developed by researchers in specially funded or university-based situations may not be well implemented by social workers or clinicians who are usually required to implement the program in the context of a complex array of sociopolitical realities. Since school administrators and teachers are the “primary adopters” of such programs, their support is essential for the continuation of prevention programs within the school setting. As shown in Flannery and Torquati's [10] research, “teachers who are not satisfied with a program are less likely to use the program materials, regardless of whether their principal or district administration is supportive of the program” (p. 395). Furthermore, an increasing number of researchers have recently advocated that program evaluation should not only assess the merit of a program's past performance, but also factors that will help the program staff to improve program implementation in the future [11, 12]. Obviously, program implementers have a particular role in providing their opinions regarding the activities being implemented and their suggestions on how the program can be improved. Based on the views of program implementers, program managers/researchers can make better decisions about how to adjust the program strategies and activities. Hence, in evaluating as well as monitoring the implementation of a school-based program, the views of the program implementers must be taken into account.

Nevertheless, there are several arguments against the use of evaluation data collected from the program implementers. The first argument is that program implementers may not have the required expertise in conducting the evaluation. Second, there may be role strain and role confusion involved if program implementers have to perform the roles of both program implementer and evaluator. Third, there are several sources of bias that are involved in the evaluation conducted by program implementers. In the first place, because program implementers have to be accountable for their delivered service, they may boost the effectiveness rating for the sake of job security (i.e., rice-bowl argument). In addition, because program implementers have invested time and effort in the program implementation process, it is difficult for them to evaluate a program in a negative manner (i.e., cognitive dissonance argument). On the other hand, because the program implementers may not be totally willing to implement a program, they may consciously or unconsciously minimize the program effectiveness and evaluate the program in an unfair manner (i.e., revenge argument).

However, there are several counterarguments responding to the above criticisms of involving program implementers in the evaluation process. First, some professionals (such as teachers and social workers) are trained to conduct evaluation research. Second, because evaluation is part of the practice in many professions, professionals are actually expected to implement the program as well as to evaluate the program. In the case of teachers and social workers, role conflict is basically not a problem. In fact, they are expected to carry out both program implementation and evaluation tasks in their practice. In addition, the emphasis on reflective practice in these professions actually encourages professionals to evaluate the delivered programs in an honest and sincere manner. Third, based on different evaluation perspectives (e.g., qualitative evaluation, illuminative evaluation, utilization-focused evaluation), it is legitimate and indispensable to collect the views of the program implementers (conservative view) or to engage the program implementers as evaluators (liberal view).

The Project P.A.T.H.S. (Positive Adolescent Training through Holistic Social Programmes) [13–16] is a positive youth development program designed for junior secondary school students in Hong Kong. After completion of the Tier 1 Program (curricular-based program designed for Secondary 1 to 3 students), program participants and program implementers were required to complete subjective outcome evaluation forms (Form A and Form B, resp.). Based on the subjective outcome evaluation data collected from each school, the responsible worker was required to complete an evaluation report, where they were asked to write down five conclusions regarding the program and its effectiveness. In this study, secondary data analyses were carried out in order to examine the subjective outcome evaluation findings based on the program participants. The purpose of this study was to integrate research findings based on different cohorts of program implementers in the Experimental Implementation Phase and Full Implementation Phase of the project.

## 2. Methods

### 2.1. Participants and Procedures

From 2005 to 2009, the total number of schools that participated in the Project P.A.T.H.S. was 244, with 669 schools in the Secondary 1 level, 443 in the Secondary 2 level, and 215 in the Secondary 3 level. Altogether, there were 9,915 instructors who participated in the Tier 1 Program in these 5 years. The mean numbers of teachers and social workers implementing the program per school per form were 4.79 (range: 0–28) and 2.60 (range: 0–12), respectively. In these three grades, the mean number of students per school was 167.28, with an average of 4.61 classes per school. Among them, 46.27% of the respondent schools adopted the full program (i.e., 20 h program involving 40 units), whereas 53.73% of the respondent schools adopted the core program (i.e., 10 h program involving 20 units). The mean number of sessions used to implement the program was 22.77 (range: 3–66). While 51.54% of the respondent schools incorporated the program into the formal curriculum (e.g., Liberal Studies, Life Education), 48.46% used other modes (e.g., class teachers' periods and any classes that differed from the normal class schedule) to implement the program. Data characteristics can be seen in Table 1.

After completing the Tier 1 Program, the implementers were invited to respond to the Subjective Outcome Evaluation Form (Form B) developed by the first author [17]. From 2005 to 2009, a total of 7,926 questionnaires were completed (4,096 for the Secondary 1 level, 2,602 for the Secondary 2 level, and 1,228 for the Secondary 3 level). The overall response rate was 79.94%. To facilitate the program evaluation, the research team developed an evaluation manual with standardized instructions for collecting the subjective outcome evaluation data [17]. In addition, adequate training was provided to the implementers during the 20 h training workshops on how to collect and analyze the data collected by Form B.

The respondents replied to Form B in a self-report format. They were asked to indicate if they did not want to respond to the evaluation questionnaire (i.e., “passive” informed consent was obtained). Adequate time was provided for the respondents to complete the questionnaire.

### 2.2. Instruments

The Subjective Outcome Evaluation Form (Form B) was used to measure the program implementers' perceptions of the Tier 1 Program. Broadly speaking, there are several parts in this evaluation form as follows.

- Program implementers' perceptions of the program, such as program objectives, design, classroom atmosphere, interaction among the students, and the students' participation during class (10 items).
- Program implementers' perceptions of their own practice, including their understanding of the course, teaching skills, professional attitude, involvement, and interaction with the students (10 items).
- Workers' perceptions of the effectiveness of the program on students, such as promotion of different psychosocial competencies, resilience, and overall personal development (16 items).
- The extent to which the workers would recommend the program to other students with similar needs (1 item).
- The extent to which the workers would teach similar programs in the future (1 item).
- The extent to which the program implementation has helped the workers' professional growth (1 item).
- Things that the workers obtained from the program (open-ended question).
- Things that the workers appreciated most (open-ended question).
- Difficulties encountered (open-ended question).
- Areas that require improvement (open-ended question).

For the quantitative data, the implementers were requested to input the collected data into an Excel file developed by the research team that would automatically compute the frequencies and percentages associated with the different ratings for an item. When the schools submitted the reports, they were also requested to submit the soft copy of the consolidated datasheets. After receiving the consolidated data by the funding body, the data were aggregated in order to “reconstruct” the overall profile based on the subjective outcome evaluation data as collected by the research team.

### 2.3. Data Analyses

Percentage findings were examined using descriptive statistics. A composite measure of each domain (i.e., perceived qualities of program content, perceived qualities of program implementers, and perceived program effectiveness) was created based on the total scores of each domain divided by the number of items. Pearson correlation analysis was used to examine if the program content and program implementers were related to the program effectiveness. Multiple regression analysis was performed to compare which domain would predict the program effectiveness. All analyses were performed by using the Statistical Package for Social Sciences Version 17.0.

## 3. Results

Quantitative findings based on the closed-ended questions are presented in this paper. Several observations can be highlighted from the findings. First, the program implementers generally had positive perceptions of the program (Table 2), including clear objectives of the curriculum (94.06%), a strong and sound theoretical support (85.65%), and well-planned teaching activities (88.44%). Second, a high proportion of the implementers had a positive evaluation of their own performance (Table 3). For example, 98.60% of the implementers perceived that they were ready to help their students, 98.36% of the implementers expressed that they cared for the students, and 96.19% believed that they had good professional attitudes. Third, as shown in Table 4, many implementers perceived that the program promoted the development of students, including their social competence (92.17%), self-understanding (92.13%), moral competence (90.34%), and overall development (92.43%). Fourth, 87.90% of the implementers would recommend the program to students with similar needs. Fifth, 80.83% of the implementers expressed that they would teach similar courses again in the future. Finally, 82.05% of the respondents indicated that the program had helped their professional development (Table 5).

Reliability analyses with the schools as the unit of analysis showed that Form B was internally consistent (Table 6): 10 items related to the program (*α* = 0.94), 10 items related to the implementer (*α* = 0.92), 16 items related to the benefits (*α* = 0.97), and 36 items measured program effectiveness overall (*α* = 0.98). Results of correlation analyses showed that both program content (*r* = 0.78, *P* < 0.01) and program implementers (*r* = 0.65, *P* < 0.01) were strongly associated with program effectiveness (Table 7).

Table 8 presents multiple regression analysis results. Higher positive views toward the program and program implementers were associated with higher program effectiveness (*P* < 0.01). Further analyses showed that perceived program content (*β* = 0.66) was a significantly stronger predictor than program implementers (*β* = 0.37). This model explained 91% of the variance toward the prediction of program effectiveness. Interestingly, the above relationships and the amount of variance were consistent across grade levels.

## 4. Discussion

The purpose of this study was to integrate the evaluation findings of the Tier 1 Program of the Project P.A.T.H.S. based on the perspective of the program implementers. There are several unique features of this study. First, in contrast to the common focus on the program participants alone, the present study examines subjective outcome evaluation findings based on several cohorts of students. Second, a large sample involving 244 schools and 7,926 participants was utilized in the present analysis. Third, responses from students in different grades in the junior secondary school years were collected. Fourth, this is the first known scientific subjective outcome evaluation study in different Chinese communities. Finally, it is also a rare attempt in the international literature on positive youth development that examines subjective outcome evaluation as derived from the program implementers' perspective.

Generally speaking, the quantitative findings showed that a high proportion of the workers had positive perceptions of the program and themselves; roughly four-fifths of the respondents regarded the program as helpful to the program participants. The findings are basically consistent with those findings reported previously based on the perspective of the program implementers in the Experimental and Full Implementation Phases using separate cohorts as the bases of analysis. In fact, an examination of the percentages of responses to different items revealed that the figures were very similar across different studies. Furthermore, the findings are generally consistent with those findings based on subjective outcome evaluation data collected from the program participants.

One of the unique things about the present study is the involvement of the program implementers as evaluation partners throughout the evaluation process. Researchers noted the importance of active participation of the program stakeholders for enhancing the use of evaluation findings [18, 19]. The program stakeholder could be viewed as “valid local data” ([20]; p. 92) because they have relevant information and knowledge that is valuable to the program evaluation process but is not known by the program evaluators. They act like program experts who are able to identify program attributes that should be addressed and evaluate work effectively due to their diversified roles, such as administration, management, and operations, during the program implementation process [21]. By utilizing their expertise and practical knowledge, the program will better match with local needs and therefore increase the validity of evaluation findings. This practice is a constructive response to Guba [22], who emphasized the establishment of local nature of an evaluation process and defined evaluation as “a local process with its outcomes depends on local contexts, local stakeholders, and local values” (p. 40). With the involvement of program implementers, the quality and credibility of the program evaluation findings would be enhanced [6].

Another advantage of the involvement of program stakeholders is the promotion of their evaluation capacity and engagement in the program. During the evaluation process, stakeholders are more motivated to design an appropriate program, respond to the changes quickly, and play a greater role in modifying the program towards meeting the needs of the program participants. In particular, they would utilize their evaluative skills and knowledge effectively, integrate and apply what they learned from evaluation data, and become more responsive to the participants' concerns. Through this ongoing reflection process, their sense of ownership and dedication towards the program would be fostered [23, 24]. This is critical, especially when the role of the principal researchers and evaluators will gradually diminish once the funds are depleted. In short, the practice of engaging program implementers in the evaluation process can enhance the motivation and commitment of the program implementers.

There are three strengths of this study. First, the subjective outcome evaluation findings are based on a large sample size (*n* = 7, 926 workers involving 244 schools). Such a big sample size substantially enhances the generalizability of the research findings to other student populations. Second, different aspects of subjective outcome, including views of the program, worker, perceived effectiveness, and overall satisfaction, were covered in the study. Third, the present study demonstrates the strategy of “reconstructing” the overall profile of the subjective outcomes based on the reports submitted by the participating schools. In fact, this study is the first published scientific study utilizing this “reconstruction” approach based on such a large number of workers in a series of databases in the Chinese culture.

However, there are several limitations of the study. First, because the data were reconstructed from the reports submitted by the schools; the unit of analysis was schools rather than individual program participants. As such, characteristics at the individual level cannot be examined. Second, while the reconstructed profile can give some ideas about the global picture, those unfavorable responses were diluted. Future study should examine such unfavorable responses qualitatively.

Third, although it is possible to interpret the positive findings in terms of program success, it is noteworthy that there are several alternative explanations of these findings. The first alternative explanation is the “beauty in the eye of the beholder” hypothesis. Because the workers are the stakeholders and they are personally involved in implementing the program, they tend to look at the program effect and their own performance in a more favorable light. The second alternative explanation is the “cognitive dissonance” hypothesis. Because the workers may have beliefs about the value of the program, it would be difficult for them to rate the program and themselves in an unfavorable manner. In particular, negative evaluation would pose a threat to the professional self and self-esteem of the workers. The third alternative explanation is the “survival” hypothesis, which maintains that the positive subjective outcome evaluation findings occurred as a result of the participants' anxiety that the program would be terminated if the evaluation findings were not positive. This possibility can be partially dismissed because the funding body has never linked funding to program success and there is no league table in the evaluation findings. The final alternative interpretation is that the workers may consciously respond in a “nice” manner to help the researchers illustrate positive program effect. However, this alternative explanation could be dismissed because negative ratings were recorded (e.g., whether the workers would teach similar courses again) and the workers responded in an anonymous manner. Despite these limitations, the present findings suggest that the Tier 1 Program and its implementation were perceived in a positive manner by the program implementers and the workers perceived the program to be beneficial to the development of the students and the program implementers. In view of the limited international and local research studies documenting the perceptions of workers in youth development or prevention programs, the present study can be regarded as a useful contribution.

## Figures and Tables

**Table 1:** Description of data characteristics from 2005 to 2009.

	S1				S2			S3	
	2005/2006 EIP	2006/2007 FIP	2007/2008 FIP	2008/2009 FIP	2006/2007 EIP	2007/2008 FIP	2008/2009 FIP	2007/2008 EIP	2008/2009 FIP
Total schools that joined P.A.T.H.S.	52	207	213	197	49	196	198	48	167
(i) 10 h program	23	95	108	104	27	113	110	29	104
(ii) 20 h program	29	112	105	93	22	83	88	19	63

Tier 1 Program									

Mean number of sessions of program implementation	17.75 (3–50)	23.55 (2–50)	23.61 (5–60)	23.54 (5–65)	23.76 (10–40)	22.81 (7–60)	23.04 (4–48)	24.07 (10–44)	22.78 (7–66)
Number of schools incorporated into formal curriculum	21	101	116	98	26	108	99	30	85
Number of schools incorporated into other modes	31	106	97	99	23	88	99	18	82
Mean number of classes per school	4.58 (2–7)	4.66 (1–8)	4.69 (1–8)	4.56 (1–8)	4.51 (1–7)	4.62 (1–8)	4.64 (1–8)	4.56 (1–8)	4.67 (1–8)
Mean number of students per school	166.90 (37–240)	172.63 (17–280)	171.05 (16–267)	158.78 (5–251)	166.67 (32–240)	170.66 (12–280)	169.61 (15–263)	160.58 (26–240)	168.60 (28–240)
Total number of instructors	419	1582	1630	1458.5	336	1486	1473.5	344	1186
Mean number of teachers per school	5.13 (0–17)	5.47 (0–14)	5.63 (0–28)	5.75 (0–28)	2.27 (0–6)	5.59 (0–15)	5.63 (0–20)	2.25 (0–6)	5.40 (0–20)
Mean number of social workers per school	2.63 (0–8)	2.13 (0–9)	2.00 (0–8)	1.75 (0–10)	4.55 (0–12)	1.97 (0–8)	1.76 (0–10)	4.90 (0–12)	1.68 (0–7)
Total number of instructor respondents	344	1250	1324	1178	270	1178	1154	286	942
Mean number of instructor respondents per school	6.62 (1–21)	6.04 (1–18)	6.22 (1–29)	5.98 (1–24)	5.51 (2–15)	6.01 (1–17)	5.83 (1–16)	5.96 (1–18)	5.64 (1–16)

S1: Secondary 1 level; S2: Secondary 2 level; S3: Secondary 3 level; EIP: Experimental Implementation Phase, FIP: Full Implementation Phase.

**Table 2:** Summary of the program implementers' perceptions towards the program.

		Respondents with positive responses (options 4–6)							
		S1		S2		S3		Overall	
		*n*	%	*n*	%	*n*	%	*n*	%
(1)	The objectives of the curriculum were very clear	3,865	94.45	2,437	94.02	1,149	93.72	7,451	94.06
(2)	The design of the curriculum was very good	3,416	83.52	2,144	82.94	1,031	84.09	6,591	83.52
(3)	The activities were carefully planned	3,634	88.87	2,289	88.48	1,076	87.98	6,999	88.44
(4)	The classroom atmosphere was very pleasant	3,564	87.40	2,182	84.38	1,009	82.77	6,755	84.85
(5)	There was much peer interaction among the students	3,516	86.18	2,174	84.20	1,009	83.18	6,699	84.52
(6)	Students participated actively during lessons (including discussions, sharing, games, etc.)	3,496	85.88	2,104	81.65	974	80.30	6,574	82.61
(7)	The program had a strong and sound theoretical support	3,496	86.02	2,180	84.86	1,043	86.06	6,719	85.65
(8)	The teaching experience I encountered enhanced my interest in the course	3,234	79.60	2,010	78.39	953	78.76	6,197	78.92
(9)	Overall speaking, I have a very positive evaluation of the program	3,222	78.99	2,033	78.71	948	78.15	6,203	78.62
(10)	On the whole, students liked this curriculum very much	3,236	79.57	1,969	76.67	920	75.85	6,125	77.36

*Note: *All items are on a 6-point Likert scale with 1 = strongly disagree, 2 = disagree, 3 = slightly disagree, 4 = slightly agree, 5 = agree, 6 = strongly agree. Only respondents with positive responses (options 4–6) are shown in the table.

**Table 3:** Summary of the program implementers' perceptions towards their own performance.

		Respondents with positive responses (options 4–6)							
		S1		S2		S3		Overall	
		*n*	%	*n*	%	*n*	%	*n*	%
(1)	I had a good mastery of the curriculum	3,507	86.38	2,212	86.44	1,016	84.53	6,735	85.78
(2)	I was well prepared for the lessons	3,563	88.13	2,262	88.60	1,030	85.83	6,855	87.52
(3)	My teaching skills were good	3,567	88.71	2,226	88.23	1,024	86.63	6,817	87.86
(4)	I have good professional attitudes	3,901	96.61	2,444	96.26	1,139	95.71	7,484	96.19
(5)	I was very involved	3,804	94.16	2,367	93.04	1,085	91.18	7,256	92.79
(6)	I gained a lot during the course of instruction	3,410	84.70	2,132	83.90	986	83.14	6,528	83.91
(7)	I cared for the students	3,990	98.66	2,501	98.35	1,171	98.07	7,662	98.36
(8)	I was ready to offer help to students when needed	4,000	98.99	2,512	98.66	1,173	98.16	7,685	98.60
(9)	I had much interaction with the students	3,759	93.09	2,331	91.74	1,086	91.11	7,176	91.98
(10)	Overall speaking, I have a very positive evaluation of myself as an instructor	3,876	95.77	2,389	94.02	1,119	94.03	7,384	94.61

*Note: *All items are on a 6-point Likert scale with 1 = strongly disagree, 2 = disagree, 3 = slightly disagree, 4 = slightly agree, 5 = agree, 6 = strongly agree. Only respondents with positive responses (options 4–6) are shown in the table.

**Table 4:** Summary of the program implementers' perceptions towards the program effectiveness.

		Respondents with positive responses (options 3–5)							
		S1		S2		S3		Overall	
		*n*	%	*n*	%	*n*	%	*n*	%
The extent to which the Tier 1 Program (i.e., the program in which all students have joined) has helped your students									
(1)	It has strengthened students' bonding with teachers, classmates, and their families.	3,674	90.36	2,268	88.28	1,055	87.05	6,997	88.56
(2)	It has strengthened students' resilience in adverse conditions.	3,495	85.98	2,196	85.55	1,038	85.79	6,729	85.77
(3)	It has enhanced students' social competence.	3,795	93.31	2,356	91.67	1,102	91.53	7,253	92.17
(4)	It has improved students' ability in handling and expressing their emotions.	3,675	90.41	2,258	87.86	1,049	86.62	6,982	88.30
(5)	It has enhanced students' cognitive competence.	3,465	85.30	2,173	84.75	1,019	84.28	6,657	84.78
(6)	Students' ability to resist harmful influences has been improved.	3,409	83.88	2,151	83.76	990	81.89	6,550	83.18
(7)	It has strengthened students' ability to distinguish between the good and the bad.	3,708	90.90	2,324	90.11	1,098	90.00	7,130	90.34
(8)	It has increased students' competence in making sensible and wise choices.	3,553	87.15	2,231	86.61	1,051	86.15	6,835	86.64
(9)	It has helped students to have life reflections.	3,372	82.99	2,162	84.26	1,040	85.95	6,574	84.40
(10)	It has reinforced students' self-confidence.	3,337	82.07	2,036	79.31	939	77.67	6,312	79.68
(11)	It has increased students' self-awareness.	3,817	93.67	2,361	91.65	1,110	91.06	7,288	92.13
(12)	It has helped students to face the future with a positive attitude.	3,429	84.11	2,136	82.92	1,021	83.76	6,586	83.60
(13)	It has helped students to cultivate compassion and care about others.	3,458	85.13	2,191	85.39	1,016	83.83	6,665	84.78
(14)	It has encouraged students to care about the community.	3,174	78.10	2,019	78.62	935	77.21	6,128	77.98
(15)	It has promoted students' sense of responsibility in serving the society.	3,168	77.93	2,016	78.75	933	77.11	6,117	77.93
(16)	It has enriched the overall development of the students.	3,815	93.62	2,375	92.13	1,116	91.55	7,306	92.43

*Note: *All items are on a 5-point Likert scale with 1 = unhelpful, 2 = not very helpful, 3 = slightly helpful, 4 = helpful, 5 = very helpful. Only respondents with positive responses (options 3–5) are shown in the table.

**Table:** (a) If you have a student/client whose needs and conditions are similar to those of your students who have joined the program, will you suggest him/her to participate in this program?

Respondents with positive responses (options 3–4)							
S1		S2		S3		Overall	
*n*	%	*n*	%	*n*	%	*n*	%
3,609	89.49	2,232	87.36	1,038	86.86	6,879	87.90

*Note: *The item is on a 4-point Likert scale with 1 = definitely will not suggest, 2 = will not suggest, 3 = will suggest, 4 = definitely will suggest. Only respondents with positive responses (options 3–4) are shown in the table.

**Table:** (b) If there is a chance, will you teach similar programs again in the future?

Respondents with positive responses (options 3–4)							
S1		S2		S3		Overall	
*n*	%	*n*	%	*n*	%	*n*	%
3,335	83.21	2,013	79.94	933	79.34	6,281	80.83

*Note: *The item is on a 4-point Likert scale with 1 = definitely will not teach, 2 = will not teach, 3 = will teach, 4 = definitely will teach. Only respondents with positive responses (options 3–4) are shown in the table.

**Table:** (c) Do you think the implementation of the program has helped you in your professional growth (e.g., enhancement of your skills)?

Respondents with positive responses (options 3–5)							
S1		S2		S3		Overall	
*n*	%	*n*	%	*n*	%	*n*	%
3,319	82.56	2,098	82.50	974	81.10	6,391	82.05

*Note: *All items are on a 5-point Likert scale with 1 = unhelpful, 2 = not very helpful, 3 = slightly helpful, 4 = helpful, 5 = very helpful. Only respondents with positive responses (options 3–5) are shown in the table.

**Table 6:** Means, standard deviations, cronbach's alphas, and mean of interitem correlations among the variables by grade.

	S1		S2		S3		Overall	
	M (SD)	*α* (Mean^#^)	M (SD)	*α* (Mean^#^)	M (SD)	*α* (Mean^#^)	M (SD)	*α* (Mean^#^)
Program content (10 items)	4.38 (0.42)	0.94 (0.59)	4.34 (0.45)	0.95 (0.65)	4.33 (0.49)	0.95 (0.67)	4.36 (0.45)	0.94 (0.62)
Program implementers (10 items)	4.65 (0.30)	0.91 (0.51)	4.63 (0.34)	0.93 (0.58)	4.61 (0.38)	0.94 (0.63)	4.64 (0.33)	0.92 (0.56)
Program effectiveness (16 items)	3.33 (0.37)	0.97 (0.66)	3.33 (0.39)	0.97 (0.69)	3.33 (0.43)	0.98 (0.71)	3.33 (0.39)	0.97 (0.68)
Total effectiveness (36 items)	3.99 (0.33)	0.97 (0.50)	3.97 (0.35)	0.98 (0.53)	3.96 (0.40)	0.98 (0.58)	3.98 (0.35)	0.98 (0.52)

*Note:* All ANOVA results were not significant.

^#^Mean interitem correlations.

**Table 7:** Correlation coefficients among the variables.

Variable		1	2	3
(1)	Program content (10 items)	—		
(2)	Program implementers (10 items)	0.71**	—	
(3)	Program effectiveness (16 items)	0.78**	0.65**	—

**P < 0.01.

**Table 8:** Multiple regression analyses predicting program effectiveness.

	Predictors			
	Program content	Program implementers	Model	
	*β* ^ a^	*β* ^ a^	*R*	*R* ^2^
S1	0.68**	0.36**	0.96	0.91
S2	0.63**	0.39**	0.95	0.90
S3	0.66**	0.35**	0.96	0.92
Overall	0.66**	0.37**	0.95	0.91

^a^Standardized coefficients.

**P < 0.01.
